# Microbial Metabolites in the Maturation and Activation of Dendritic Cells and Their Relevance for Respiratory Immunity

**DOI:** 10.3389/fimmu.2022.897462

**Published:** 2022-07-08

**Authors:** Kayla R. Wilson, Elise Gressier, Malcolm J. McConville, Sammy Bedoui

**Affiliations:** ^1^ Department of Microbiology and Immunology at the Peter Doherty Institute for Infection and Immunity, University of Melbourne, Parkville, VIC, Australia; ^2^ Department of Biochemistry and Pharmacology, Bio21 Institute of Molecular Science and Biotechnology, University of Melbourne, Melbourne, VIC, Australia

**Keywords:** microbiota, dendritic cells, metabolites, respiratory, immunity

## Abstract

The respiratory tract is a gateway for viruses and bacteria from the external environment to invade the human body. Critical to the protection against these invaders are dendritic cells (DCs) - a group of highly specialized myeloid cells that monitors the lung microenvironment and relays contextual and antigenic information to T cells. Following the recognition of danger signals and/or pathogen molecular associated patterns in the lungs, DCs undergo activation. This process arms DCs with the unique ability to induce the proliferation and differentiation of T cells responding to matching antigen in complex with MHC molecules. Depending on how DCs interact with T cells, the ensuing T cell response can be tolerogenic or immunogenic and as such, the susceptibility and severity of respiratory infections is influenced by the signals DCs receive, integrate, and then convey to T cells. It is becoming increasingly clear that these facets of DC biology are heavily influenced by the cellular components and metabolites produced by the lung and gut microbiota. In this review, we discuss the roles of different DC subsets in respiratory infections and outline how microbial metabolites impact the development, propensity for activation and subsequent activation of DCs. In particular, we highlight these concepts in the context of respiratory immunity.

## Introduction

Respiratory infections claim millions of lives each year and as such represent one of the top ten causes of death worldwide (1). Their threat to human health has recently been underscored by the coronavirus disease (COVID-19) pandemic which, in addition to damaging the global economy, has led to the death of more than six million people (2). Many components of the innate and adaptive immune system respond to the diverse set of pathogens that infect the upper and lower airways. Of particular interest are dendritic cells (DCs), a highly specialized myeloid cell type that plays a key role in regulating diverse functions of T cells in clearing respiratory infections and maintaining long-lasting immunity as well as inducing pathology.

DCs are derived from the bone marrow (BM) and *via* the blood populate virtually all tissues, including mucosal sites of the upper and lower respiratory tract. In such barrier tissues, they continuously monitor the microenvironment for antigens, danger signals and other cues reflective of the local context. To communicate these local cues to naïve CD8^+^ and CD4^+^ T cells that are restricted in their circulation to primary and secondary lymphoid organs, DCs migrate *via* lymphatic vessels from peripheral tissues to the local draining lymph nodes (dLNs). Upon arrival in the T cell zone, DCs present antigen to T cells *via* major histocompatibility complex class I (MHC I) and class II (MHC II) molecules. While only those T cells expressing matching T cell receptors (TCRs) are activated in response to such antigen presentation, it is the provision of additional signals by the DCs that ultimately determines whether the ensuing T cell response is tolerogenic or immunogenic (3).

Danger signals (DAMPs) and/or pathogen-associated molecular patterns (PAMPs) stimulate DCs to undergo a complex differentiation program that is often referred to as DC activation. Such DCs display antigen in the context of increased levels of costimulatory molecules and the secretion of a variety of soluble mediators. The accessory signals are highly context dependent and dictate the activation and acquisition of effector functions by the responding T cells. For example, T cell responses against Herpes simplex virus (HSV) depend on DC-produced interleukin (IL)-15 (4), whereas T cell responses against *Leishmania major* require DC-produced IL-12 (5). The induction of specific T cell responses therefore reflects the amalgamation of various environmental cues received and integrated by DCs.

Although long thought of as a sterile environment, it is now clear that the respiratory tract is inhabited by diverse microbiota and many findings suggest that the presence of the microbiota and, more importantly, their metabolites heavily influence DC function and thus impact T cell immunity against many respiratory pathogens. This review focusses on the current understanding of how microbial metabolites, both locally and possibly remotely, shape DC functions. In particular, we discuss how microbiota-derived metabolites impact the ability of DCs to sense and respond to innate signals and how they in turn communicate contextual cues associated with an infection to T cells.

## Respiratory Tract DCs

The lungs are continuously exposed to the external environment which provides a myriad of microbe-derived antigens. Given DCs harbor pattern recognition receptors (PRRs) capable of recognizing these MAMPs and PAMPs, it is unsurprising that the respiratory tract is home to an elaborate network of these cells. During steady-state, three DC subsets reside in the respiratory tract; migratory CD103^+^ DCs, migratory CD11b^+^ DCs and plasmacytoid DCs (pDCs) (6–8). The migratory CD103^+^ and CD11b^+^ DCs comprise of cells analogous to conventional dendritic cell (cDC) subset 1 (cDC1) and subset 2 (cDC2) in terms of their function and origin. In addition, they comprise monocyte-derived DCs (moDCs) (9, 10) which outnumber their conventional counterparts during inflammatory conditions (11).

During steady-state, cDCs are the most common DC in the lung (7). cDCs are released from the BM as precursors (pre-cDCs) which undergo further development to cDC1s and cDC2s in peripheral tissues (12, 13). The egress of pre-cDCs from the BM to the lungs requires CC-chemokine receptor type 2 (CCR2) and CX3 chemokine receptor 1 (CX3CR1), with reduced number of pre-cDCs and cDCs observed in the lungs of mice lacking these receptors (14). In contrast, pDCs are fully developed prior to leaving the BM and migrate to the lungs in a CCR5 (15) and CCR2 (16) dependent manner. Inflammation following the inhalation of lipopolysaccharide (LPS) (14) or infection (7, 11, 13) increases the number of pre-cDCs, cDCs and moDCs in the lungs. DC lung infiltration during infection is dependent on CCR2 with reduced infiltration observed in mice lacking this receptor (11, 14, 17).

## CD103^+^ DCs

In the respiratory tract, migratory CD103^+^ DCs are identified by their expression of CD11c, CD103, CD207 (Langerin) and X-C motif chemokine receptor 1 (XCR1). The human equivalent are CD141^+^ BDCA-3^+^ DCs (18). Their development depends on the transcription factors basic leucine zipper transcription factor 3 (BATF3), inhibitor of DNA protein 2 (ID2) and interferon (IFN) regulatory protein 8 (IRF8) in addition to signaling *via* FMS-like tyrosine kinase 3 (Flt3) (19, 20). As such, mice lacking BATF3, ID2, IRF8 or Flt3 have a reduced number of CD103^+^ DCs (19, 20). Some CD103^+^ DCs are also derived from Ly6C^hi^ CCR2^hi^ monocytes (9, 10), although their lineage relationship to cDC1s remains less well defined. CD103^+^ DCs reside in the mucosa of the trachea and large conducting airways, in close proximity to the epithelial cells (7, 21, 22). They express tight junction proteins, such as claudin-1 and -7, which allow them to extend their dendrites through the basolateral space and sample antigen in the airway lumen (7, 23). CD103^+^ DCs are also found in the lung interstitium (22, 24, 25).

Following antigen acquisition and DC activation, migratory CD103^+^ DCs traffic to the dLNs in a CCR7-dependent manner (26–28). Here, they present antigen to CD8^+^ and CD4^+^ T cells *via* MHC I and II. In addition to canonical antigen presentation, CD103^+^ DCs are specialized in the cross-presentation of cell-associated antigen (27, 29, 30). Indeed, cross-presentation of intranasally inoculated cell-associated antigen is lost in *Batf3^-/-^* mice lacking CD103^+^ DCs, in addition to resident CD8^+^ DCs (29). This is particularly important for the clearance of viral respiratory infections. For example, influenza A virus X-31 (H3N2) or PR8 (H1N1) infected mice lacking migratory CD103^+^ DCs (langerin-diphtheria toxin receptor, DTR, or Clec9A-DTR mice treated with DT) have significantly increased viral burden and weight loss in comparison to untreated infected mice (21, 31). Furthermore, reduced influenza-specific CD8^+^ T cells are observed (21, 31). Similar findings are observed for bacterial respiratory infections, such as those caused by *Mycobacterium tuberculosis* (32) or *Bordetella pertussis* (33). Given DT treatment ablates all DCs, an important question is whether the locally activated CD103^+^ DCs are responsible for CD8^+^ T cell activation. To determine this, GeurtsvanKessel and colleagues (21) selectively depleted respiratory DCs by administering DT intratracheally and notably found that local CD103^+^ DCs indeed played a significant role in the clearance of influenza.

In addition to presenting antigen directly to CD8^+^ T cells, migratory CD103^+^ DCs also contribute to the capacity of resident cDC1s to activate CD8^+^ T cells. Resident mediastinal lymph node (mLN) cDC1s from influenza A infected mice stimulate CD8^+^ T cells when cultured *ex vivo* (21, 30, 34). Given antigen is not easily detected in these cells, it is thought they acquire it from other cells. This has been elegantly demonstrated with the use of transporter associated with antigen processing 1 (TAP1) deficient CD103^+^ DCs that are unable to carry out MHC I antigen presentation. Indeed, when incubated with *Tap1* ^-/-^ CD103^+^ DCs from infected mice, resident cDC1s from uninfected mice stimulate antigen-specific CD8^+^ T cells *in vitro* (30). Since only the resident cDC1s are capable of priming CD8^+^ T cells in this system, it demonstrates that migratory DCs relay antigen to resident cDC1s. This idea of antigen transfer is strongly supported by other models of viral infection, such as HSV type 1 (HSV-1) skin infection (35). During the early phases of infection, CD8^+^ T cell priming is carried out by LN-resident cDC1s, not migratory DCs (36, 37). However, this priming still requires migratory DCs, with impaired CD8^+^ T cell priming observed when DC migration is ablated (36). Together with the finding that direct infection of resident cDC1s is not required for effective CD8^+^ T cell priming and control of the infection (38), migratory DCs indeed appear to be a key source of antigen for cDC1s to present to CD8^+^ T cells. Importantly, migratory CD103^+^ DCs can cross-present to CD8^+^ T cells in certain scenarios. For example, CD103^+^ DCs can stimulate CD8^+^ T cells during the later stages of skin HSV-1 infection (39), or when the route of infection is changed from the skin to the lungs (30). What environmental cues cause migratory DCs to behave differently during different stages or routes of infection remains a curiosity. It is possible that they can acquire antigen from other migratory DCs just as resident cDC1s and that their relative sparsity in LNs at early stages of the infection makes any meaningful cross-presentation difficult to detect experimentally.

## CD11b^+^ DCs

Migratory CD11b^+^ DCs are another major DC population in the respiratory tract. They are identified by their expression of CD11c, CD11b, CX3CR1 and SIRPα in mice, or CD1c (BDCA-1) in humans (18). Steady-state lung CD11b^+^ DCs comprise of pre-cDCs and monocyte-derived cells, with Ly6C^lo^CCR2^lo^ monocytes contributing to the latter (9). The development of pre-cDC derived CD11b^+^ DCs depends on IRF4, with mice lacking IRF4 having a reduced number of lung CD11b^+^ DCs (40). During inflammation, the greatest contributor to respiratory tract CD11b^+^ DCs are monocytes. CD11b^+^ DCs are found within the submucosa of the conducting airways and the lung interstitium (7, 21, 22). Classically, cDC2s play important roles activating CD4^+^ T cells *via* MHC II antigen presentation. Once activated, CD4^+^ T cells differentiate into helper T (Th) cells which can promote B cell antibody responses and CD8^+^ T cells responses - both of which are important for the clearance of respiratory infections (41). In addition to their helper functions, Th cells can directly provide protection against infection. For example, studies have demonstrated that adoptively transferred naïve or effector CD4^+^ T cells differentiate into Th1 cells, migrate to the site of infection and release high levels of IFN-γ (42), a cytokine with key roles in clearing intracellular infections. A similar phenotype is observed for adoptively transferred influenza-specific memory CD4^+^ T cells (43). Here, CD4^+^ T cell production of IFN-γ reduces viral titers and provides protection against secondary infections. This is also observed for other viral respiratory infections, such as Sendai virus (44). Independent of IFN-γ secretion, CD4^+^ T cell cytotoxicity has been demonstrated during viral infection, particularly lethal doses of influenza (45). CD4^+^ T cell cytotoxicity is still controversial, however, with the exact mechanism and physiological relevance much less explored than that of CD8^+^ cytotoxicity. Since CD11b^+^ DCs are well equipped to activate CD4^+^ T cells, it is unsurprising that when isolated from LNs of mice intratracheally injected with ovalbumin (OVA)-Cy5 they stimulate CD4^+^ T cells to a greater extent than CD103^+^ DCs *in vitro* (27). Similar results are observed with CD11b^+^ DCs isolated from mice infected with influenza A or respiratory syncytial virus (RSV) (46, 47). It is worthwhile noting, however, that CD4^+^ T cells primed by CD11b^+^ DCs do appear to require a second hit from cDC1s to gain full effector function (48), thus raising the prospect that cDC1s not only cross-present antigen but also provide additional signals to CD4^+^ T cells that are critical for their differentiation.

CD11b^+^ respiratory tract DCs can act as sources of antigen for LN-resident CD8^+^ DCs (21). It has also been suggested that CD11b^+^ respiratory tract DCs can cross-present viral antigen to CD8^+^ T cells, though the biological relevance of this is questionable given their efficiency is less than that of cDC1s. CD11b^+^ respiratory tract DCs traffic to dLN with viral antigen where they outnumber CD103^+^ DCs (21, 46, 47, 49). When isolated *in vitro*, these DCs can stimulate antigen-specific CD8^+^ T cells (47, 49) in a CD70-dependent manner (49). Cross-presentation by CD11b^+^ DCs requires inflammatory conditions, as it is not observed when mice are administered non-infectious influenza virion (47). The mechanism, however, is unclear with some studies suggesting the cells may be infected or simply cross-dressing (29, 50). Regardless, it is unlikely that CD11b^+^ DC cross-presentation is critical for viral immunity given they cannot compensate for the loss of cDC1s (20, 26, 29, 51).

## pDCs

pDCs express high levels of Siglec-H and bone marrow stromal antigen-2 (BST-2) and low levels of CD11c. The human counterparts are BDCA-2^+^ CD123^+^ (18). The development of pDCs depends on E2-2 (52, 53) and loss of this transcription factor leads to reduced numbers of pDCs (52, 53). Similar to CD11b^+^ DCs, pDCs are found in the large conducting airways and lung interstitium (22, 24, 54). pDCs are well known for their antiviral responses due to their high expression of toll-like receptor (TLR) 7, TLR9, retinoic acid-inducible gene I (RIG-I) and melanoma differentiation-associated protein 5 (MDA5). These PRRs allow pDCs to recognize single-stranded and double-stranded RNA in addition to unmethylated CpG motifs commonly found in viral DNA. Once recognized, pDCs respond by producing high levels of type I and III interferons (IFN-I and -III) (55). The role of pDCs in respiratory infections is context dependent. During infection with influenza A/PR8 virus, pDCs are the major producers of IFN-α and, during the early stages of infection, promote lymphocyte recruitment to the respiratory tract (56). They can also traffic to mLNs with high levels of viral antigen. While inefficient at priming T cells, they may act as source of antigen during infection (21). Even so, the loss of this subset does not impact immunity against influenza. Indeed, mice lacking pDCs (*Ikaros^L/L^*) (56) or depleted of pDCs (120G8 mAb) (21) clear influenza virus to the same extent as wild type mice. Unlike influenza, pDCs appear to play a role in the clearance of RSV. RSV-infected pDC-depleted mice have reduced IFN-α and elevated lung viral titers and pulmonary inflammation (57–59), suggesting this subset in particular is important for RSV protection. Interestingly, IFN-α treatment only rescues the elevated lung viral titers (57), suggesting during RSV, pDCs prevent lung inflammation *via* an IFN-α-independent mechanism. In agreement with this, altered T cell responses are observed in RSV-infected mice depleted of pDCs (57, 59, 60) which cannot be rescued by IFN-α treatment (57). How exactly pDCs perform this function is currently unclear. pDCs are also important for promoting regulatory T cell (Treg) responses during RSV, but only in neonates. Tregs are specialized CD4^+^ T cells which suppress immune responses through a variety of mechanisms including producing inhibitory cytokines, such as IL-10, and suppressing DCs (61). In particular, Tregs play an important role in controlling lung inflammation and immunopathology in RSV or pneumonia virus of mice (PVM) infection (62–64). Neonatal mice lacking pDCs have increased disease severity. This phenotype depends on pDC-expressed semaphorin-4a (Sema4a), which promotes Treg proliferation, and can be rescued by adoptive transfer of Tregs (62). Importantly, this is specific to neonates with regular Treg expansion observed in RSV-infected pDC-depleted adult mice (62). Therefore, by producing IFN-α and supporting T cell responses in adults and in neonates, pDCs prevent severe infection and lung inflammation during RSV.

All together, these studies demonstrate that DCs play a critical role in the susceptibility and severity of respiratory infections. They do this by trafficking to T cell zones during infection, where they prime CD4^+^ and CD8^+^ T cells and promote cytotoxic T cell activity. Migratory DCs also promote the activation of T cells indirectly by acting as sources of antigen for resident DCs, which in turn promote effector and memory T cell responses. Further to this, DCs can effectively protect against excessive inflammation and tissue damage by promoting the expansion of tolerogenic T cells. While these roles are well established, a number of questions still remain unanswered. Firstly, given DCs are continuously sampling the environment without inducing immunogenic T cell immune responses, how are they able to rapidly shift to different activation states during an infection? That is, what determines the activation threshold for DCs and in turn allows them to carry out tolerogenic or immunogenic functions? Secondly, how do DCs integrate multiple signals to determine the T cell response? For example, why does the cross-presenting ability of CD103^+^ DCs differ depending on the site or duration of infection? Similarly, what environmental cues sway pDCs from promoting lung inflammation during some infections, such as influenza, and preventing it during other infections, such as RSV? Finally, since DCs are short-lived, it would be remiss not to question the role DC progenitors play in inadvertently shaping T cell responses by controlling the generation of DCs at steady-state and during infection. Unlike what is observed during steady-state, in infected lungs, DCs derived from monocytes far exceed the number of DCs that are derived from pre-cDCs. It is therefore important to understand what environmental cues DC progenitors receive in the BM and how they integrate these cues to determine the output of DCs. In particular, the role of microbiota-derived metabolites in controlling immune cell function and responses in the gut and lung has garnered considerable attention.

## Gut and Lung Microbiota

Under selection pressure from the host immune system, coevolution can lead to the emergence of more virulent pathogen strains with potentially superior immune evasion strategies. However, co-evolution can also have beneficial outcomes as is the case for the human microbiota. In this relationship, the microbiota promotes and supports important physiological functions, such as the digestion of macromolecules and provision of essential nutrients (for example vitamins) which the host is incapable of. In return, the host provides a nutrient-rich environment in which microbes can thrive. The gut microbiome has been most intensively studied in humans and comprises approximately 10^13^ bacteria (65), in addition to viruses, fungi and archaea. Most microbes reside in the colon, with healthy individuals housing over 100 bacterial species here. The most abundant classes and species are those belonging to the Firmicutes and Bacteroidetes phyla (66–68). Microbial communities are also found in the upper and lower airways (69–73). The presence of microbiota in the lungs was only empirically confirmed by bacterial DNA sequencing in 2010/11 (72, 74) and as such, studies investigating the function of the lung microbiota have lagged those focused on the gut. This is mainly due to the outdated dogma of lung sterility, invasive sampling requirements (for example, bronchoalveolar lavage) and risks of contamination during sampling due to low bacterial burden in the healthy lung (75). Nevertheless, it is now well established that the lung harbors a dynamic microbiota. Like the gut, Bacteroidetes and Firmicutes phyla predominate the healthy lung. The most frequently observed families include *Prevotellaceae*, *Veillonellaceae* and *Streptococcaceae* (69, 71–73, 76, 77).

The stability of the gut and lung microbiota can be impacted by a number of factors, including infection, inflammation, diet and antibiotic exposure. The human microbiota is known to influence host innate and adaptive immune cells, and dysbiosis can have severe consequences for the susceptibility and outcome of respiratory infections. The microbiota communicates with immune cells locally or systemically *via* its cell components or secreted metabolic end-products. Cell components include LPS, lipoproteins, lipoteichoic acid (LTA), peptidoglycan (PG), CpG motifs, flagellin and cyclic dinucleotides (CDN). These molecules are sensed by TLRs (78), nucleotide-binding and oligomerization domain (NOD)-like receptors (NLRs) (79), C-type lectin receptors (CLRs) (80) and cyclic-GMP-AMP (cGAS)/stimulator of interferon genes (STING) (81) expressed in cells throughout the body. In brief, binding of ligands to TLRs leads to the recruitment of MyD88 and activation of NF-κB and IRF-5 signaling pathways which induce the transcription of proinflammatory cytokines (78). Recognition of ligands by some TLRs, such as LPS by TLR4 or CpG by TLR9, can also activate IRF-7 or IRF-3 signaling pathways which promote transcription of IFN-α/β (78). NF-κB-dependent induction of proinflammatory cytokines is also observed when NLRs are stimulated by PG (79). Alternatively, NLR stimulation can induce inflammasome formation which leads to the post-translational cleavage of proinflammatory cytokines, such as pro-IL-18 (79). When CDNs are recognized by cGAS/STING, subsequent IRF-3 and NF-κB activation induces the transcription of type I interferons and proinflammatory cytokines (81). As such, microbiota have multiple ways through which they can stimulate the immune system, and this appears to have substantial pathophysiological relevance as discussed further below.

The various constituents of the microbiota also secrete a variety of metabolites following catabolism of dietary and/or host-derived carbohydrates (e.g mucins), proteins, lipids and bile acids (BAs). Accordingly, many studies have focused on understanding the link between diet and microbial-derived metabolites, host cell metabolism and immune function. The best-studied metabolites are short-chain fatty acids (SCFAs), such as acetate (C2), propionate (C3) and butyrate (C4), which are the product of anaerobic fermentation of non-digestible dietary fiber. Acetate and propionate are mainly produced by bacteria belonging to Bacteroidetes phylum whereas butyrate is predominately produced by bacteria belonging to Firmicutes phylum (82, 83). Measuring the concentration of SCFAs in the colon and other organs of humans is difficult given the invasive sampling methods that are required. A study of sudden death victims demonstrated that SCFAs are present in the colon at a concentration of 20-140 mM (approximate molar ratio of 60:20:20) (84). Most studies, however, infer SCFA concentrations from fecal content, with secretion rates estimated between 5–30 mmol/day depending on fiber intake (85). Butyrate is utilized as a major carbon source by colonocytes, and together with other SCFAs also enters the portal circulation for transport to the liver and other organs. Therefore, fecal concentrations are not a true indication of SCFA production rates (85). SCFAs can be detected in portal, hepatic and peripheral blood (84, 86, 87). The highest concentrations are observed in portal blood with concentrations of acetate, propionate and butyrate around 240-262 µM, 30–88 µM and 26–29 µM, respectively (84, 86, 87). Importantly, some of these values may be inflated by failure to adjust for background levels of acetate observed in many mass spectrometry assays. SCFA levels drop in the peripheral blood to approximately 10-80 µM (acetate), 0.4-20 µM (propionate) and 0.2-2µM (butyrate), while levels of isobutyrate, 2-methylbutyrate and isovalerate are generally <0.5µM (84, 87). Given the capacity to produce SCFAs varies between bacteria found in the gut, the concentration of SCFAs varies depending on diet. Indeed, increases in portal and peripheral SCFAs can be observed as quickly as 15 minutes following lactulose cecal instillation (87). Measurements of SCFAs in peripheral organs is controversial. Indeed, some murine studies have indicated that SCFA cannot be detected in the lungs (88), while others have detected low µM concentrations (89).

SCFAs can cross cellular membranes by diffusion or be taken up *via* Na^+^ or proton-coupled solute carrier family 5A8 (SLC5A8/SMCT1) (90, 91) and 16A1 (SLC16A1/MCT1) (92) proteins. Once inside the cell, SCFAs are catabolized *via* enzymes in the fatty acid β-oxidation pathway to generate acetyl-CoA which can drive mitochondrial oxidative phosphorylation (OXPHOS), fatty acid biosynthesis and protein acetylation. While not extensively studied in DCs, 13^C^-butyrate is incorporated into tricarboxylic acid cycle (TCA) intermediates in T cells (93). Functionally, this leads to increased oxygen consumption and glycolysis ultimately contributing to stemness and longevity of CD8^+^ T cells, thus promoting their differentiation into memory cells. Additionally, propionate and butyrate, but probably not acetate, inhibit histone deacetylases (HDAC) 1 and 3 (94). This in turn regulates acetylation of histones and transcription factors thereby altering gene expression. SCFA-induced HDAC inhibition has an array of physiological consequences depending on the cell type and scenario. For example, it can promote apoptosis in neutrophils (95) or the differentiation of highly antibacterial macrophages (96). SCFAs can also activate cell surface G protein-coupled receptors (GPRs), including GPR41 (also known as free fatty acid receptor 3, FFAR3), GPR43 (FFAR2), GPR109a and olfactory receptor 78 (Olfr78) (97). GPR41 and GPR43 recognize all three SCFAs, whereas GPR109a binds to butyrate only. Olfr78 binds acetate and propionate (98–101). DCs express GPR41, 43 and 109a (102–104) while the expression of Olfr78 is currently unclear. Activation of these GPRs can induce a number of intracellular signal transduction pathways depending on the G proteins coupled. GPR41, 43 and 109a are coupled to G_i_, which promotes the cyclic adenosine monophosphate (cAMP) and phospholipase C (PLC)/protein kinase C (PKC)/extracellular signal regulated kinase (ERK) pathways (97). GPR43 can also couple with G_q_ proteins leading to the activation of PLC/inositol trisphosphate (IP3) pathway and the release of Ca2^+^ (97). SCFA-induced activation of GPRs can have wide-ranging impacts on immunity. For example, GPR41/43 activation alters BM hematopoiesis in allergic airway inflammation (88), promotes memory CD8^+^ T cell responses in HSV infection (93) and neutrophil infiltration in influenza infection (102). Moving forward, it will be important for studies to elucidate the underlying mechanisms of how GPR activation can have such diverse outcomes and resolve the relative importance of different means through which SCFA can influence and enter a cell.

Another major group of metabolites produced by the gut microbiota are secondary BAs. Primary BAs, such as cholic acid (CA) and chenodeoxycholic acid (CDCA), are synthesized *via* cholesterol catabolism in hepatocytes (105, 106), conjugated with glycine or taurine, and stored in the gall bladder before being secreted into the small intestine upon food ingestion. BAs facilitate the absorption of lipids before undergoing deconjugation in the distal small intestine. BA deconjugation is a common process carried out by a wide array of bacteria belonging to all the major bacteria phyla (107, 108). BAs are either reabsorbed and transported back to the liver or excreted in the feces. Alternatively, the gut microbiota can convert BAs to secondary BAs, such as lithocolic acid (LCA) and deoxycholic acid (DCA), *via* the 7α-dehydroxylation pathway (108, 109). This pathway is encoded by the *bile acid inducible* (*bai*) operon, which is expressed in only a few members of the *Clostridium* and *Eubacterium* genera (108, 109). The cecal concentration of BAs in recently deceased individuals is 0.43 mM, with LCA and DCA representing the predominant BAs (110). Primary and secondary BAs can reenter the circulation and regulate immune responses at distant organs by binding to BA activated receptors (BARs) expressed on hematopoietic and nonhematopoietic cells. BARs include the nuclear receptors (NRs) farnesoid x receptor (FXR), vitamin D receptor (VDR) and pregnane-x-receptor (PXR); and the GPR Takeda G-protein receptor 5 (TGR5/GPBAR1). Importantly, as all of these are expressed on DCs (106, 111–113), secondary BAs have the potential to regulate DC responses at steady-state and during infection. FXR preferentially binds to CDCA in humans and CA in mice, whereas DCA and LCA are the preferential ligands for TGR5, though they can also activate VDR and PXR (106). Once activated, BARs regulate the transcription of target genes that are involved in a plethora of physiological processes including lipid, glucose and energy metabolism (105, 106). For example, activation of TRG5 in DCs and macrophages inhibits NF-κB-dependent proinflammatory cytokine expression (113, 114).

Gut microbial metabolites can also regulate cholesterol metabolism and uptake from the digestive tract (115), as well as affecting cholesterol metabolism in some monocyte-derived immune cells. Specifically, some gut microbes convert dietary PC, choline and carnitine to triethylamine (TMA), which is transported to the liver and converted to trimethylamine N-oxide (TMAO) by hepatic monooxygenase-3. So far, eight bacterial species have been shown to produce TMA: *Anaerococcus hydrogenalis, Clostridium asparagiforme, Clostridium hathewayi, Clostridium sporogenes, Escherichia fergusonii, Proteus penneri, Providencia rettgeri* and *Edwardsiella tarda* (116). TMAO levels in the serum are linked to diet (particularly high intake of meat, eggs, or fish) and directly correlate with a number of diseases, including atherosclerosis and chronic kidney disease (117–119). TMAO induces increased expression of scavenge receptors (CD36, SRA-1) in monocytes and macrophages. This leads to increased uptake of oxidized lipoproteins and reduced cholesterol efflux (118). It also promotes the formation of foamy macrophages that contribute to the deposition of atherosclerotic plaques in arteries (118). TMAO increases the expression of IL-6 and tumor necrosis factor (TNF) in macrophages, which further promotes a proinflammatory environment and atherosclerosis plaque formation (120). The extent to which TMAO regulates lipid metabolism in DCs in other tissues has not been investigated.

In addition to having important roles in carbohydrate and BA catabolism, gut commensals also play an important role in the metabolism of proteins. While the majority of dietary protein is digested and absorbed in the small intestine, a small amount transits to the colon (121) where it can be further broken down by the microbiota. Released aromatic amino acids (tryptophan, tyrosine and phenylalanine) are converted to indolic acid and phenolic compounds (122) that regulate gastrointestinal integrity locally. Notably, they also exert systemic effects on immune cells after reentering circulation. For example, *p*-cresol sulfate (PCS), a product of L-tyrosine catabolism, impacts the recruitment and activation of DCs to the lungs and alleviates allergic airway inflammation (123). Investigations into how aromatic amino acid metabolites are recognized and integrated by host cells are still in their infancy. Indeed, while some metabolites, such as the tryptophan derivatives indole propionic acid (IPA) and indole-3-pyruvic acid (IPyA), have been shown to activate aryl hydrocarbon receptor (Ahr) (122), further studies are required to uncover cognate receptors for other metabolites and their level of promiscuity. Besides aromatic acid catabolism, gut commensals also contribute to the production of arginine derived metabolites, such as the polyamines putrescine, spermidine and spermine (124). Bronchoalveolar lavages have demonstrated that polyamines are present in the lungs and that their concentration is altered in asthmatic patients (125), suggesting they may play a role in lung immunity. In DCs, polyamines exert their effect by regulating signaling pathways. For example, DCs incubated with spermidine have increased activation of Src kinase, indoleamine 2,3-dioxygenase 1 (IDO1) (126) and Forkhead box protein O3 (FOXO3) (127). In addition, impaired NF-κB activation is observed (127). Together, these changes correlate with a more immunosuppressive phenotype (125–127). Indeed, impaired secretion of cytokines and upregulation of activation markers, such as CD86, is observed in DCs incubated with spermidine (125, 127). Since polyamines are charged, entry into cells requires transport proteins (124), however, it is currently unclear which proteins are responsible for entry into DCs. Furthermore, investigations are required to determine the bacteria that are responsible for the production of amino acid catabolites in healthy and infected individuals. Given amino acid metabolism occurs in both the host and microbiota, it is important for future studies to delineate the contribution of each to host immunity.

It should be noted that since specific bacteria are responsible for the release of specific metabolites or components, the composition of the microbiota has considerable influence on the availability of metabolites and as a result, immunity. An example of this is the gram-negative bacterial cell component LPS which is either penta-acylated or hexa-acylated. The former is produced in *Lpxl*-expressing gram-negative bacteria (such as Bacteroidetes) and is less inflammatory. The latter is produced by gram-negative bacteria expressing both *Lxpl* and *Lpxm* (such as γ-proteobacteria), and is highly inflammatory. Metagenomic analysis has shown that while LPS-producing bacteria are prevalent in healthy lungs, the majority produce the less-inflammatory penta-acylated form (128). In contrast, elevated hexa-acylated LPS producing bacteria are observed in asthmatic patients (128). While further work is required to determine if this is a causal relationship, it does highlight how the composition of the microbiota, and as a result the composition of metabolites, can potentially skew immune responses.

An important question that remains to be answered is whether respiratory tract immune cells receive these microbiota-derived signals from the local environment or if they are derived from the gut *via* the circulation. Some metabolites, such as SCFA and secondary BAs, may only be produced in the gut due to the nature of their synthesis. Therefore, the ability of these metabolites to penetrate peripheral tissues is an important factor to consider given they cannot impact respiratory DC function if they are unable to reach DC progenitors in the BM or DCs in the respiratory tract or LNs. Other components, such as LPS however, can potentially be derived from both the lung and gut microbiota. This has recently been demonstrated *via* intratracheal neomycin administration, which skews the lung microbiota composition towards LPS-producing bacteria without impacting the composition of the gut microbiota (129). Importantly, this shift impairs central nervous system immune responses which in turn alleviates symptoms in a model for lung experimental autoimmune encephalomyelitis (129). It is therefore likely that both the respiratory and gut microbial metabolites are relevant *in vivo*. In agreement with this, during influenza infection, intranasal or intrarectal administration of TLR ligands can restore immunity in antibiotic treated mice (130). Furthermore, dysbiosis of the gut microbiota is often observed in respiratory infections, such as tuberculosis (131). Similarly, gastrointestinal illnesses, such as inflammatory bowel disease, increase susceptibility to respiratory infections (132). Together these studies demonstrate bidirectional communication between the gut and lung microbiota, perhaps even suggesting a level of meta-organization of the microbiomes that seemingly exist and operate in isolation at distinct anatomical sites.

Bacterial components or metabolites may also regulate DCs indirectly *via* other cells. While there are limited studies demonstrating this in the lungs, the crosstalk between epithelial cells and DCs is well-established in the gut. For example, microbiota derived SCFAs have been shown to promote the expression of retinaldehyde dehydrogenase 1 (RALDH1) in intestinal epithelial cells. This promotes retinoic acid production which in turn acts on intestinal CD103^+^ DCs to promote tolerogenic phenotypes (133). Given CD103^+^ DCs are in close contact with respiratory epithelial cells, it will be interesting to determine if, like their intestinal counterparts, they are indirectly regulated by lung microbiota-derived metabolites.

## Impact of Microbial Metabolites on DC Development

The mechanism in which microbial metabolites impact respiratory DC function is currently unclear. There have been suggestions it may involve alterations of BM hematopoiesis and thus DC development (88), which in turn influences what type of DCs populate the airways and lungs. Despite this, splenic and lymph node DC numbers and frequencies are unaltered in GF mice in comparison to specific pathogen free (SPF) mice (130, 134, 135). Therefore, the loss of the microbiota does not impact the steady-state generation of DCs. However, as detailed in subsequent sections, this does not mean the DCs generated in the absence of microbiota are fully functional. In contrast, boosting microbiota-derived signaling, as observed by treating cells or mice with SCFAs, promotes the generation of DC progenitors and impacts the development of DCs in a context-dependent manner.

*In vitro*, butyrate and propionate impair the development of DCs from murine BM cells or human monocytes (**Table 1**). Specifically, murine BM cells cultured in the presence of granulocyte-macrophage colony-stimulating factor (GM-CSF) and butyrate or propionate are impaired in their differentiation into CD11c^+^ cells compared to cells cultured in the absence of SCFAs (94). Similar results are observed during the differentiation of moDCs from human monocytes (136, 137). This phenotype depends on SLC5A8 and GPR109A, with improved, but not totally restored BMDC differentiation observed in cells lacking either of these receptors (94). Butyrate and propionate are thought to impair *in vitro* DC differentiation by regulating transcription factors necessary for the development of DCs *via* HDAC inhibition (94), though more thorough genome-wide transcription and chromatin accessibility studies are required to understand the full mechanisms at play. Importantly, these studies utilized GM-CSF for DC development instead of Flt3 ligand (Flt3L). DCs developed in these cultures are developmentally and phenotypically distinct, with the former aligning more closely to inflammatory DCs and the latter to steady-state DCs (147, 148). As such, it is unclear whether SCFA treatment impacts the development of Flt3L-dependent DCs *in vitro*. This is an important question with regards to high-fiber diets and their impact on the immune system, given that high-fiber diets increase the concentration of SCFA by up to 100-fold (102).

**Table 1:** Microbial metabolites and their impact on DCs *in vitro* and *in vivo*.

Metabolite	Effect *in vitro*	Effect *in vivo*	Reference
Butyrate and propionate	↓ DC development ↓ upregulation of costimulatory molecules ↓ proinflammatory cytokine secretion ↓ chemokine expression ↓ allostimulatory capabilities ↑ *RALDH1* expression ↑ H4 acetylation ↑ *Ido1* expression ↑ Tr1 cell induction ↑ Treg cell induction	↑ Development of immunosuppressive DCs during inflammation ↑ immunosuppressive phenotype ↓ Th2 cell induction ↓ airway inflammation	(88, 94, 104, 136–142)
DCA	↓ upregulation of costimulatory molecules ↓ proinflammatory cytokine secretion ↓ NF-κB activation	↑ ISG expression ↑ CHIKV protection	(113, 143, 144)
LPS		↑ DC migration ↑ influenza protection	(130)
Spermidine	↑ *Ido1* expression ↑ p-Src expression ↑ FOXO3 expression ↓ NF-κB activation ↓ upregulation of costimulatory molecules ↓ proinflammatory cytokine secretion	↑ immunosuppressive phenotype ↓ airway inflammation ↑ lung function	(125–127)
PCS		↓DC lung infiltration during airway inflammation ↓DC activation during airway inflammation ↓DC migration during airway inflammation	(123)
DAT		↑ ISG expression ↑ influenza protection	(145)
?		↑ ISG expression ↑ cDC poised for infection	(134, 146)

**Figure 1:**
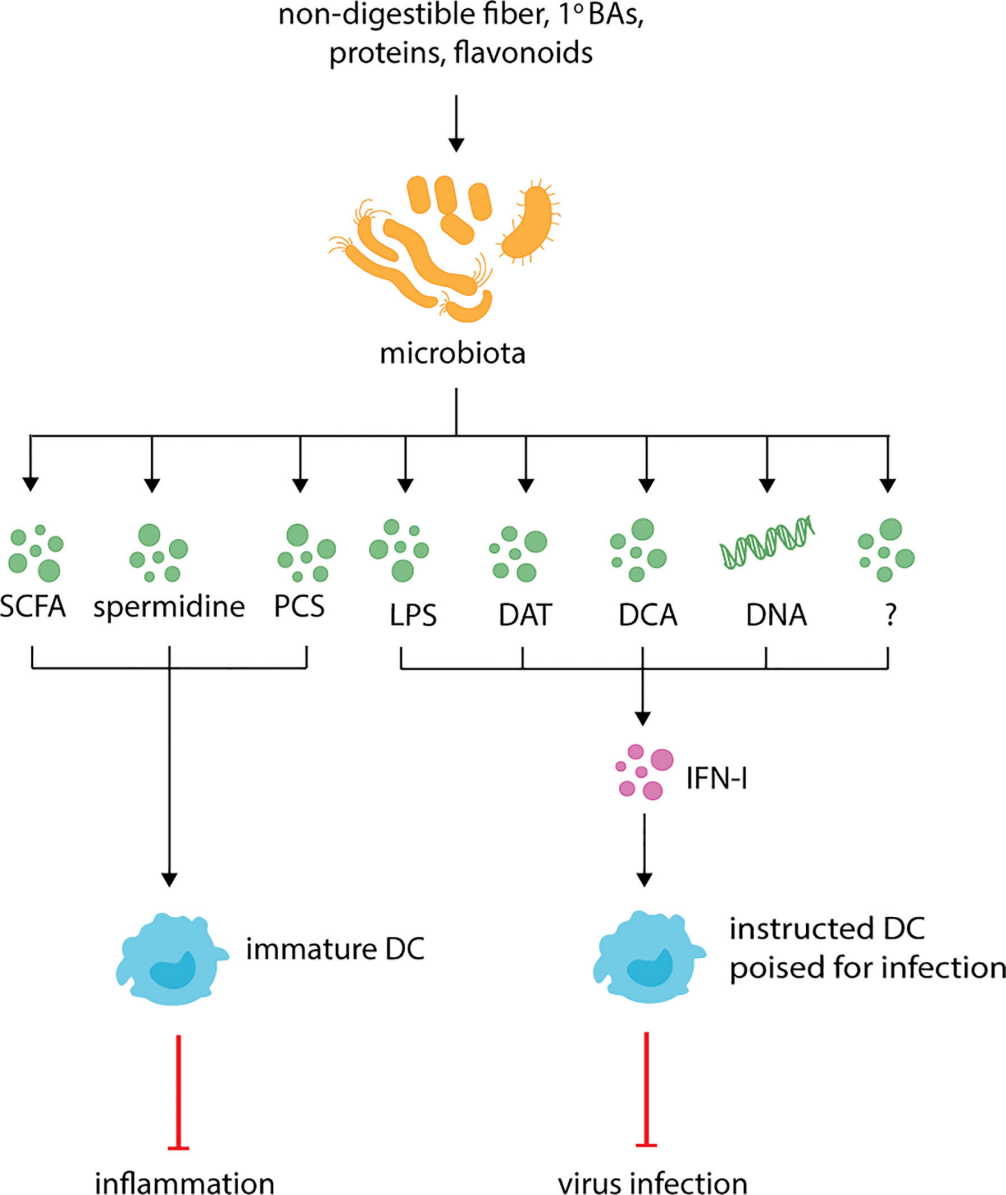
Microbiota derived metabolites regulate DC function and respiratory health. The microbiota produces known metabolites and cell components such as short-chain fatty-acids (SCFAs), *p*-cresol sulfate (PCS), desaminotyrosine (DAT), deoxycholic acid (DCA), lipopolysaccharide (LPS) and currently uncharacterized metabolites (indicated by the question mark). During inflammation, SCFAs, spermidine and PCS dampen inflammation by promoting the generation of functionally immature DC which dampen Th2 responses. At steady-state, LPS, DAT, DCA, microbiota-derived DNA and potentially other uncharacterized metabolites instruct DCs for future immune responses by promoting the production of IFN-I.

In contrast to steady-state conditions, SCFAs promote the generation of DC BM progenitor cells during inflammatory conditions *in vivo* (**Figure 1**, **Table 1**). House dust mite exposed mice treated with propionate have increased common DC progenitors (CDPs) and macrophage-DC progenitors (MDPs), promoting the generation of moDCs (88). Increased BM progenitors (MDPs) are also observed when mice are fed a high-fiber diet (102). However, during influenza infection, this promotes the production of Ly6C^-^ monocytes which give rise to alternatively activated macrophages, not DCs. Interestingly, even though the outcome of microbiota-altered hematopoiesis is highly context dependent, both studies demonstrate a reliance on the expression of GPR41. It will be important to uncover how the host integrates similar microbiota-derived signals with inflammatory signals to generate context-dependent hematopoietic outcomes.

Microbiota-dependent hematopoiesis promotes anti-inflammatory responses during respiratory diseases and infections (**Figure 1**, **Table 1**). As described above, during allergic inflammation airway diseases, the expanded progenitor populations observed in mice fed a high-fiber diet promote the influx of newly generated moDCs to the lungs. However, these DCs are phenotypically less activated and have altered Th2 priming capabilities that correlate with reduced inflammation (88). Similarly, during influenza infection, the high-fiber diet-induced expansion of alternatively activated macrophages reduces inflammatory neutrophil recruitment to lungs and prevents severe immunopathology (102).

**Figure 2:**
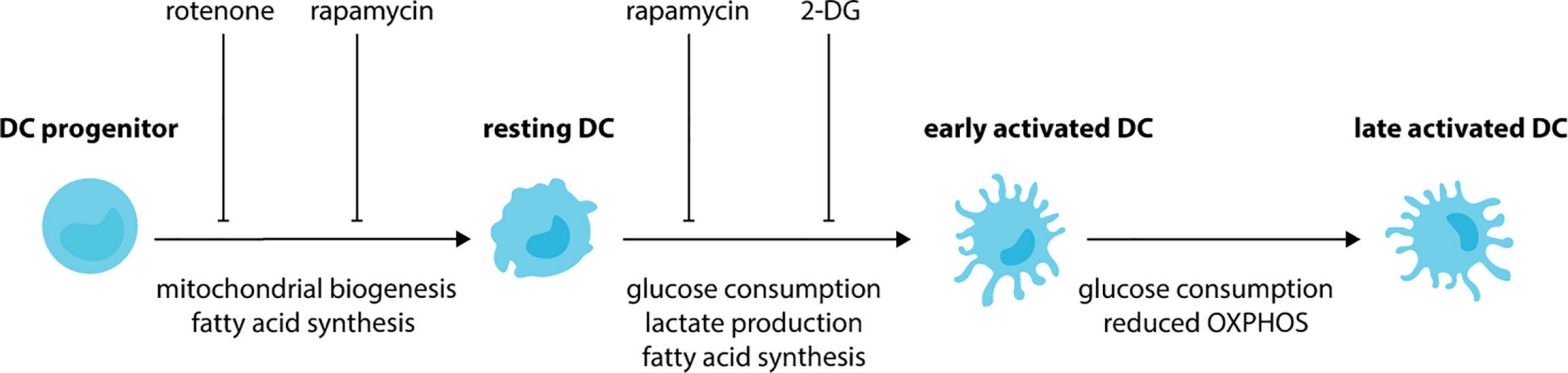
DCs undergo metabolic rewiring during development and activation. During the development of DCs, progenitor cells increase mitochondrial biogenesis and fatty-acid synthesis. These metabolic changes lead to DCs with an increased mitochondrial metabolism in comparison to their progenitors. Treatment of progenitor cells with rotenone (respiratory chain inhibitor), TOFA (acetyl CoA carboxylase inhibitor) and rapamycin (mTORC1 inhibitor) impairs the development of DCs. Upon exposure to activating stimuli, resting DCs rapidly increase consumption of glucose and their production of lactate and fatty acids. As a result, impaired activation is observed when DCs are treated with glycolysis inhibitor 2-DG, fatty-acid synthase inhibitor C75 or TOFA. Glycolysis is maintained during the later stages of DC activation, however, a reduction in OXPHOS also occurs.

SCFAs may impact the development of DCs by remodeling metabolic networks in progenitor cells (149). During the differentiation of DCs from progenitor cells there is an increase in mitochondrial biogenesis (**Figure 2**). As such, DCs have elevated mitochondrial DNA, biogenesis-associated genes, respiratory complex proteins and ATP production in comparison to progenitor cells (150, 151). This phenotype is inhibited by the respiratory chain inhibitor rotenone, which also impairs DC development (150). Besides mitochondrial biogenesis, the development of DCs depends on fatty acid synthesis with impaired development observed when murine BM cells or human monocytes are cultured in the presence of acetyl CoA carboxylase inhibitor TOFA (152), or mammalian target of rapamycin (mTOR) complex 1 (mTORC1) inhibitor rapamycin (153–155). Together, these studies demonstrate that DC development requires OXPHOS and fatty acid synthesis. As a result, DCs have increased mitochondrial metabolism in comparison to their progenitors. Recent work has revealed that that the metabolic state of cDC1s and cDC2s differs. Compared to cDC2s, cDC1s have higher mitochondrial mass, mitochondrial membrane potential, OCR and extracellular acidification rate (ECAR) suggesting that they are metabolically more active (156–158). These differences are also associated with differences in their dependency on specific signaling cascades, with cDC1s being highly dependent on Flt3L-dependent PI3K/mTOR (154) and Hippo/Mst signaling cascades (158). Therefore, future studies should focus on the impact of microbial metabolites on the activation of signaling pathways critical for the development of DC subsets.

## Impact of Microbiota-Derived Metabolites on DC Activation

### DC Activation

DC activation is associated with further metabolic rewiring (**Figure 2**). Following BMDC exposure to activating stimuli, such as LPS, CpG or polyinosinic:polycytidylic acid (poly I:C) there is a rapid (<6 hours) increase in the extracellular acidification rate (ECAR), glucose consumption and lactate production with no change in oxygen consumption rate (OCR) (159–161). This suggests during the early stages of activation, DCs increase glycolysis. Indeed, DCs incubated in the presence of the glycolysis inhibitor 2-deoxyglucose (2-DG) have impaired activation, with reduced upregulation of MHC and costimulatory molecule. Furthermore, they are unable to prime T cells to the same extent as DCs activated in the absence of 2-DG (159). Fatty acid synthesis is also essential during the initial activation of DCs, with impaired activation observed in DCs treated with TOFA or the fatty-acid synthase inhibitor C75 (159). During the later stages of DC activation, glycolysis is maintained, however, there is a relative reduction in OXPHOS. As such, increased glycolytic rate, glucose consumption and lactate production, and reduced oxygen consumption are observed in BMDCs following stimulation with LPS for 18 hours (161, 162). Similar findings are observed for human CD1c^+^ moDCs stimulated with TLR agonists (163) or infected with influenza or rhinovirus *in vitro* (164). TLR-dependent induction of glycolysis is linked to activation of the PI3K-mTORC1 signaling cascade (162) and antagonized by activation of adenosine monophosphate activated protein kinase (AMPK) and liver kinase 1 (LKB1/STK11) (162, 165). DCs lacking LKB1 (157, 165) have increased expression of activation receptors and cytokines and increased glycolytic metabolism, demonstrating these kinases are important for restraining DC activation during steady-state. The switch to glycolysis is critical for activation. Exposure to low glucose or glycolytic inhibitors impairs BMDC activation and the upregulation of costimulatory molecules, chemokine receptors and secretion of proinflammatory cytokines (159, 162, 164, 166). This leads to reduced antigen-specific T cell priming (159, 162). Using live imaging, it has been demonstrated that glucose decreases the time taken for LPS to activate DCs, whereas glycolytic inhibitors increase activation times (161). Furthermore, migration towards chemokines, such as CCL21, is dependent on DCs shifting towards glycolysis following activation. Indeed, LPS-activated BMDCs have increased expression of CCR7, the receptor for CCL21, when exposed to glucose (159, 161). As a result, *in vitro* LPS-activated DCs treated with glycolytic inhibitors have impaired migration *in vitro* (159, 161) and *in vivo* (161). Together, these studies demonstrate that activated DCs depend on glycolysis. Furthermore, in addition to impairing CCR7 oligomerization in activated DCs, reduced glucose availability also impairs CCR7 oligomerization in resting DCs (161). This suggests that DC migration, irrespective of maturation status, may also depend on glycolysis.

SCFAs can fuel mitochondrial metabolism, raising the possibility that they directly suppress DC activation and function by remodeling central carbon metabolism. In agreement with this hypothesis, butyrate and propionate repress LPS-induced upregulation of CD40, CD80 and CD86 in GM-CSF derived BMDCs (138, 139) (**Table 1**). A similar phenotype is observed with moDCs (104, 136, 137, 140–142), although it is unclear if butyrate impacts the upregulation of MHC II and CD86 in moDCs with some studies showing no change (104, 141, 142) and others showing impaired upregulation (136, 137, 140). Butyrate may also impact the LPS-induced upregulation of proinflammatory cytokines, with reduced secretion of IL-12 (104, 136, 140–142), TNFα (136) and IL-6 (104, 141) observed when moDCs are activated in the presence of butyrate. It is unclear if butyrate affects secretion of anti-inflammatory cytokines, such as IL-10, with some studies showing moDC secretion of IL-10 is unchanged (136), increased (140) and reduced (142). These differences may be due to the stimuli tested (for example LPS and IFN-γ (136) versus LPS alone (140, 142)) as well as variations in incubation periods and concentrations utilized. Transcriptional interrogation of moDCs activated in the presence or absence of butyrate or propionate has also revealed significant reductions in the expression of chemokines including CXCL9, CXCL10, CXCL11 and CCL5 (104). Expectedly, LPS-activated, butyrate-treated DCs have impaired allostimulatory capability in comparison to DCs activated in the absence of butyrate (136, 137, 140, 142). In macrophages, butyrate activates AMPK and suppresses mTOR activation, resulting in reduced glycolytic flux (96). As previously described, mTOR activation and glycolysis are required for DC activation, therefore it is critical for future studies to investigate if butyrate-dependent impaired DC activation is due to the upregulation of AMPK and/or impaired mTOR activation. In support of this hypothesis, butyrate can activate LKB1 in colorectal cancer cell lines (167), and AMPK (downstream of LKB1) in macrophages (96).

It has been suggested that the effect of SCFAs on DC activation may lead to tolerogenic DCs which promote the differentiation of immunotolerant T regulatory type 1 cells (Tr1) (142) (**Table 1**). In support of this, increased IL-10 secretion and reduced Treg transcription factors GITR, CTLA4 or FOXP3 are observed in CD4^+^ T cells incubated with SCFA-pulsed LPS-activated moDCs (142). This phenotype depends on GPR109A expression, leading to the upregulation of RALDH1 and conversion of vitamin A to retinoic acid (142). In contrast, other studies have shown that butyrate promotes DC differentiation of Foxp3^+^ Tregs and inhibits the differentiation of proinflammatory CD4^+^ T cells (IFN-y^+^) when cocultured *in vitro* (168). This is associated with increased expression of the immunosuppressive enzymes, IDO1 or aldehyde dehydrogenase (ALDH1A), which have known roles in Treg homeostasis. While not demonstrated, the authors suggest that butyrate exerts this effect *via* HDAC inhibition. Together, these studies demonstrate that while butyrate promotes an immunosuppressive phenotype in DCs, the consequence on T cell differentiation is context dependent. Given T cells are also responsive to SCFAs (169), future studies utilizing mice lacking *Slc5a8* or GPRs specifically in terminally-differentiated DCs (for example Xcr1^cre^ mice) will be important for understanding the role of SCFA on DCs and their ability to shape T cell interactions *in vivo*.

Several mechanisms may be responsible for the effects of SCFA on DC activation. Firstly, the activation of moDCs in the presence of SCFA (e.g. butyrate) leads to reduced extracellular acidification rate (ECAR) and oxygen consumption rate (OCR) (142). This phenotype is similar to moDCs activated in the presence of glycolytic inhibitors, raising the possibility that butyrate may regulate glycolysis, although it is inconsistent with the notion that SCFA uptake increases mitochondrial metabolism. Butyrate-induced metabolic changes have also been demonstrated in colonocytes (170) and T cells (93, 102), but remain to be tested in DCs. Secondly, SCFAs can impact DCs through GPRs. Butyrate has been shown to repress LPS-induced NF-κB translocation to the nucleus in murine colonocytes (98) and BMDCs (136) *via* GPR109A. Impaired NF-κB activation would account for reduced proinflammatory cytokine secretion observed in DCs stimulated with LPS in the presence of butyrate. Thirdly, SCFAs are known to regulate histone acetylation and acetylation of transcription factors by inhibiting HDACs. MoDCs incubated in presence of butyrate, and to a lesser extent propionate, have increased histone acetylation (142). When DCs are treated with HDAC inhibitors, impaired DC activation, defined as reduced upregulation of costimulatory molecules, is observed similar to when DCs are treated with butyrate (142). This suggests butyrate-induced HDAC inhibition may also contribute to impaired DC activation. Given it is currently unclear how much each of these pathways contribute to altered DC function in the presence of SCFAs, or whether they contribute at all, it will be important for future studies to investigate more closely the mechanism of action of SCFAs on DC metabolism and transcription.

While less well-studied than SCFAs, other microbial metabolites are thought to restrain DC activation (**Table 1**). BMDCs or splenic CD11c^+^ cells treated with the secondary BA DCA have impaired LPS-induced proinflammatory cytokine secretion and upregulation of costimulatory and MHC molecules (113, 143). LPS-treated BMDCs exposed to DCA have reduced phosphorylation of the NF-κB inhibitor, IκB, and nuclear p65, suggesting altered activation is due to impaired NF-κB activation. Given this phenotype is restored by treating cells with adenylate cyclase or protein kinase A (PKA) inhibitors, this inhibition is likely due to activation of cAMP-PKA pathway (113). Importantly, while loss of TGR5 restores p-IκB and p-p65 expression, it only partially rescues DC proinflammatory cytokine secretion and expression of costimulatory molecules (113). This suggests DCA may impact other pathways, although the mechanism or identity of these are currently unknown. Similarly, PCS, the microbial metabolite generated from L-tyrosine catabolism, impairs DC activation during allergic airway inflammation (**Figure 1**, **Table 1**). PCS or L-tyrosine treated mice exposed to house dust mite have reduced lung DC infiltration, DC activation and impaired DC migration to mLN (123). These changes correlate in a PCS-dependent decrease in CCL20 secretion in airways, though the mechanism is unclear. Importantly, this phenotype protects mice from allergic airway inflammation. Given members of the Bacteroidetes and Firmicutes phyla are capable of producing PCS *in vitro* (171), it will be important for future studies to uncover the bacteria responsible for *in vivo* PCS production and whether it can be manipulated to alleviate other scenarios of inflammation. Interestingly, spermidine and spermine also protect mice against allergic airway inflammation (125) (**Figure 1**, **Table 1**). Indeed, mice exposed to house dust mite and treated with spermidine or spermine exhibit reduced lung infiltration and cytokine secretion (125). DCs stimulated with these arginine catabolites *in vitro* have impaired activation as determined by reduced pro-inflammatory cytokine secretion and activation marker expression (125, 127). Given propionate treatment also protects against allergic airway inflammation (88), it will be interesting to determine if PCS, spermidine and spermine act in a similar fashion on hematopoiesis.

Together, these studies suggest that microbial metabolites of healthy individuals tend to limit the level of classical DC activation, which in turn may be associated with more tolerant effects on other immune cells, such as T cells. They also highlight a clear need for further investigations into exactly how the metabolites interact with and impact DCs, with a focus needed on the effects on DC transcriptional, signaling and metabolic profiles. This could reveal novel pathways to target to alleviate lung inflammation or boost protection against respiratory infections.

### Activation Threshold

In contrast to its immunoregulatory roles, microbial metabolites are also thought to lower the activation threshold for immune cells, also known as “instructing” (**Figure 1**, **Table 1**). This allows DCs to become activated more easily and respond to infections more efficiently. Instructing was first suggested by Ichinohe and colleagues (130) who demonstrated that antibiotic-treated mice have impaired protection against influenza A infection. This phenotype has since been recapitulated with studies demonstrating antibiotic-treated influenza A-infected mice have increased viral load, reduced cellular and humoral responses and increased mortality in comparison to untreated infected mice (130, 145, 172, 173). Similar results are observed for antibiotic-treated mice infected with chikungunya virus (CHIKV), *S. pneumoniae* or *K. pneumonia* (144, 174, 175). In influenza infection, antibiotic-induced impaired immunity correlates with reduced expression of inflammasome-related genes, including pro-IL-1β, pro-IL-18 and NLR family pyrin domain containing 3 (NLRP3), and impaired CD103^+^ DCs migration to mLN (130). The same defect in DC migration is observed in caspase-1 deficient mice, suggesting that commensals promote transcription of inflammasome-dependent cytokines which are necessary for DC migration and subsequent T cell activation. This hypothesis is strengthened by data demonstrating that antibiotic treatment does not affect inflammasome-independent infections, such as HSV-2 or *Legionella pneumophila* (130). The link to DC “instructing” was demonstrated when protection against influenza infection was restored in antibiotic-treated mice following administration of TLR agonists, such as LPS (130). Besides inflammasome activation, the microbiome also “instructs” DCs by regulating the basal expression of IFN-I (134, 144–146) (**Figure 1**).

Reduced IFN-α/β is observed in the serum of GF or antibiotic-treated mice before and after treatment with TLR agonists (134, 146, 176) or infection with the alphavirus chikungunya virus (CHIKV) (144). As a result, pDCs and CD11c^+^ DCs isolated from GF mice during steady-state conditions have reduced expression of IFN-stimulated genes (ISGs) (134, 146). Furthermore, impaired upregulation of ISGs in these cells (134, 146), and Ly6C^hi^ monocytes (144) is observed following TLR stimulation or CHIKV infection. pDCs were found to be the source of tonic IFN-I, with reduced IFN-β observed in GF mice or mice lacking pDCs in comparison to WT mice (146). It should be noted that, at steady-state, microbiota-dependent ISG induction and IFN-I production by pDCs are very low, with some groups (144) failing to detect any changes in antibiotic-treated mice. Regardless, such tonic IFN-I production has been shown to alter the transcriptional and metabolic profiles of cDCs, ultimately “poising” them for future infections (146). GF cDCs display reduced mitochondrial membrane potential and mass in addition to reduced oxygen consumption rate, indicative of reduced OXPHOS (146). As cDC1 and cDC2 have different metabolic requirements, it would be interesting to investigate the impact of tonic IFN-I on individual subsets. In particular, cDC1 have increased mitochondrial mass and oxygen consumption in comparison to cDC2 (156–158) and may therefore be more heavily impacted by loss of microbial-induced IFN-I signaling.

Together these data demonstrate that the microbiota impacts the activation threshold of DCs allowing them to respond to foreign invaders more efficiently. How exactly it does this is controversial, with studies suggesting different bacterial components or metabolites are responsible for tonic IFN-I (**Figure 1**). During steady-state, the amino acid- and flavonoid-derived metabolite desaminotyrosine (DAT) produced by *Clostridium orbiscindens* (145, 177) increases the expression of ISGs (145). Administration of DAT prior to influenza infection, but not after, restores immunity in influenza infected mice. Similar protection is observed in antibiotic-treated mice gavaged with *Clostridium orbiscindens* (145). This phenotype depends on phagocytes, with clodronate liposome treatment inhibiting the effects of DAT. Together, this suggests that influenza clearance requires *Clostridium orbiscindens*-derived DAT for IFN-I signaling and priming of phagocytes. Secondary BAs (DCA) have also been shown to promote pDC IFN-I, with reduced DCA, IFN-I and CHIKV protection observed in antibiotic-treated mice (144) (**Figure 1**). This phenotype is restored when antibiotic-treated mice are gavaged with *Clostridium orbiscindens*, implicating another member of the *Clostridium* species in the protection against viral respiratory infections. Recently, the cGas/STING pathway has been implicated in regulating tonic IFN-I (176). Mice lacking *cGas* or *Sting* have reduced basal IFN-I which is unaffected by antibiotic treatment. Furthermore, basal IFN-I levels are unaffected in mice lacking *MyD88* and *Ticam1*. This suggests that reduced IFN-I levels following antibiotic treatment is due to reduced cGas/STING activation, and not TLR activation (176). Interestingly, the authors suggest that cGas/STING activation occurs due to host cells recognizing microbiota-derived DNA which is delivered systemically *via* membrane vesicles. As proof of principle, mice inoculated with membrane vesicles isolated from *Escherichia coli* (*E. coli*) prior to infection are better protected against HSV and vesicular stomatitis virus (176). Given pDCs are thought to be essential in tonic IFN-I production (134, 146), it is important for future studies to investigate exactly how these cells are more susceptible to microbiota-derived signals. Together, these studies point to a variety of microbial factors acting on DCs directly or indirectly to regulate their propensity for activation in different settings.

DC instructing is in stark contrast to the role of microbial metabolites in preventing activation in formerly instructed DCs. This suggests that DCs require just the right amount and type of microbial stimulation to perform optimally. In this model, DC instruction and activation are not binary events that are simply turned “on” or “off” by a single factor. Instead, they require nuance and adaptation as the DC integrates multiple signals, such as those received by PRR agonists and inflammatory molecules during steady-state and infection. A major challenge in the field is to understand the consequence of simultaneous exposure of DCs (and other immune cells) to a wide variety of different microbiota- and diet-derived metabolites *in vivo*, and how the activation of different signaling and metabolic pathways by these metabolites are integrated to modulate DC phenotypes. Delineating these complex interactions will open new avenues for manipulating DC plasticity and fine-tuning T cell responses for improved immunity.

## Open Questions

1. Determining the contribution of lung and gut microbiota in the regulation of DCs and clearance of respiratory infections: To date, most studies have focused on the role of the gut microbiota on immunity. It is now clear that the lungs harbor a dynamic and rich microbiota which is likely to impact immune cells at the site of infection. Dysbiosis at one site often leads to dysbiosis, and increased susceptibility to secondary infections, at the second site. Therefore, untangling the relative contributions of different microbiota may allow the development of therapeutics that specifically target respiratory infections and immune responses.
2. Understanding the mechanism by which microbial metabolites regulate DCs: DCs are highly specialized in antigen presentation and T cell stimulation. This function requires specific transcriptional and metabolic changes during their differentiation and activation. Importantly, cDC1, cDC2 and pDCs differ in their metabolic and transcriptional profiles which endows them with their unique functional capabilities. It will be important for future studies to investigate how microbial metabolites impact DC biology and uncover the mechanism in which microbial metabolites prime, or alternatively suppress, their activation. This will reveal novel ways in which DCs can be manipulated to promote (during infection) or suppress (during autoimmunity) immune responses.
3. Investigating the combinatorial effect of multiple metabolites on DC function and immune outcomes: Current studies have focused on the impact of one microbial component or metabolite on immune cell function in mice lacking microbiota. Given this is not a physiologically relevant scenario, it will be important for future studies to investigate the combinatorial effects of metabolites on immune cells. Taking it one step further, it will also be important to incorporate signals from the nonbacterial microbiota, such as viruses, fungi and protozoa.

## Conclusion

Respiratory infections represent a significant health and economic burden that requires novel methods of vaccination or treatment to provide protection. Since their discovery, tremendous effort has gone into understanding the role of DCs, with research clearly demonstrating these cells play an important role in respiratory immunity by acting as master regulators of T cell responses. As such, DCs are a prime candidate to target for improved respiratory immune responses. For this to occur, our focus needs to shift to include an understanding of what environmental signals DCs, or their progenitors, receive prior and during respiratory infections, how they integrate these signals and finally how these signals allow DCs to shape protective T cell responses. While the microbiota is a relatively new player in this field, research already points to their components and metabolites as the missing links that connect these different facets of DC biology together. Indeed, MAMPs and microbial metabolites heavily influence not only the development of DCs but also their priming for immunity and subsequent activation, with clear consequences for respiratory immunity. The microbiota has huge therapeutic potential, especially given how easily amendable it is to manipulation by diet. Therefore, it is particularly exciting to uncover the mechanisms in which it regulates DCs.

## Author Contributions

Conceptualization: KW and SB; Writing original draft: KW and SB; Preparing figures: KW; Writing review and editing: KW, EG, and MM; Funding acquisition: SB. All authors contributed to the article and approved the submitted version.

## Funding

Our work has been supported by the National Health and Medical Research Council (NHMRC 1124815), the Australian Research Council (ARC DP210101806) and the International Training Group (IRTG 2168) funded by the German Research Council. The authors declare that their research is supported by a 350th Anniversary Research Grant from Merck KgGA. The funders were not involved in the interpretation of data, the writing of this article or the decision to submit it for publication.

## Conflict of Interest

The authors declare that the research was conducted in the absence of any commercial or financial relationships that could be construed as a potential conflict of interest.

## Publisher’s Note

All claims expressed in this article are solely those of the authors and do not necessarily represent those of their affiliated organizations, or those of the publisher, the editors and the reviewers. Any product that may be evaluated in this article, or claim that may be made by its manufacturer, is not guaranteed or endorsed by the publisher.
